# Update on the Physiopathology of Keratoconus

**DOI:** 10.3390/medsci14050579

**Published:** 2026-09-17

**Authors:** Raul Hernan Barcelo-Canton, Alejandro Rodriguez-Garcia, Enrique O. Graue-Hernandez, Jodhbir S. Mehta

**Affiliations:** 1 Tecnologico de Monterrey, School of Medicine and Health Sciences, Institute of Ophthalmology and Visual Sciences, Monterrey 66278, Mexico; 2 Instituto de Oftalmologia Fundacion Conde de Valenciana, Mexico City 06800, Mexico; 3 Ophthalmology & Visual Sciences Academic Clinical Programme (EYE-ACP), Duke-NUS Medical School, Singapore 169857, Singapore; 4 Singapore Eye Research Institute, Singapore 169856, Singapore; 5 Singapore National Eye Centre, 11 Third Hospital Ave., Singapore 168751, Singapore

**Keywords:** keratoconus, pathophysiology, oxidative stress, corneal biomechanics, extracellular matrix

## Abstract

Keratoconus (KC) is a progressive corneal ectasia characterized by stromal thinning, steepening, and biomechanical instability. Although historically considered primarily a structural disorder, current evidence supports a multifactorial pathogenesis involving complex interactions among biomechanical, molecular, cellular, inflammatory, neurobiological, and environmental mechanisms. This narrative review provides an updated overview of KC pathophysiology, integrating current evidence across these interconnected domains. Focal reductions in corneal stiffness, altered viscoelasticity, collagen disorganization, and lamellar slippage contribute to progressive deformation under physiological stress. Oxidative stress and mitochondrial dysfunction promote reactive oxygen and nitrogen species accumulation, impaired antioxidant defenses, keratocyte apoptosis, and abnormal cellular metabolism. Dysregulated extracellular matrix turnover, characterized by increased matrix metalloproteinase activity, reduced inhibitor enzymes, altered cross-linking, and aberrant growth factor signaling, further compromises stromal integrity. Chronic low-grade para-inflammation, neurotrophic imbalance, and subbasal nerve plexus alterations may amplify proteolysis and defective tissue repair. Genetic and epigenetic susceptibility interacts with environmental and behavioral modifiers. Together, these processes form pathways that converge on focal stromal weakening and cone formation. Emerging technologies, including advanced biomechanical imaging, molecular biomarkers, multi-omics approaches, and artificial intelligence, may enable earlier detection and improve risk stratification. Further understanding the pathophysiology of KC may ultimately support the development of targeted therapies aimed at modifying the underlying disease mechanisms rather than addressing the structural consequences solely.

## 1. Introduction

The cornea is a transparent avascular tissue at the front of the eye that plays a crucial role in the patient’s refractive power [1]. It consists of five organized layers that maintain its transparency and strength [2]. It is mainly composed of collagen and proteoglycans, which allow for adequate optical clarity and structure. Disruption of these components can lead to ectatic disorders such as keratoconus (KC) [2].

KC is the most common primary ectatic corneal disease. A decade after the first Global Consensus, an updated international Delphi panel of 128 experts from 12 societies across 6 continents refined this definition, characterizing KC as a primary, bilateral, typically asymmetrical corneal ectasia with progressive anterior steepening and cone-apex centered thinning as its hallmark features [3,4]. It typically affects young patients, with the diagnosis usually made during their second decade of life, and tends to progress until the third decade. If left untreated, the disease can cause severe complications like corneal blindness and scarring [5]. A global meta-analysis for KC estimated the global prevalence to be 1.38 per 1000 population [6]. However, the prevalence of KC varies according to study types and populations described. This highlights the genetic variability and the importance of environmental factors in shaping the pathophysiology of KC.

Historically, KC was viewed as a biomechanical disorder of the cornea caused by inherent tissue weakness. However, accumulating evidence over the years has shown that the pathogenesis of KC is multifactorial, involving complex interactions among various predisposing factors [7,8]. These include oxidative stress, genetic alterations, mitochondrial dysfunction, extracellular matrix (ECM) remodeling, protease-antiprotease imbalance, and cytokine signaling in genetically susceptible corneas often exposed to environmental and behavioral influences. Originally, a ‘2-hit’ hypothesis, in which a genetic predisposition is associated with an environmental event, was accepted as necessary for developing KC [9]. The “2-hit” hypothesis has been outdated as newer understanding of the development of KC arises. Currently, KC development is known as a multifactorial hypothesis, which combines genetic susceptibility, environmental triggers, inflammation of the ocular surface, or repetitive mechanical trauma as pathways to the development of KC [4,10,11]. This framework is broadly endorsed by the most recent international consensus, which further recognizes that the disease requires a multidimensional diagnostic approach integrating structural, biomechanical, cellular, and molecular data, with artificial intelligence (AI) emerging as a key enabler of this integration [4]. This complex understanding situates KC as a multifactorial disease involving molecular, genetic, cellular, biomechanical, and neurobiological processes.

Understanding these interconnected mechanisms is essential not only for comprehending the disease but also for improving risk assessment, biomarker identification, and guiding future treatment strategies. This narrative review aims to provide an updated overview of the pathophysiology of KC, integrating insights from molecular pathways, biomechanics, genetics, and environmental modifiers.

## 2. Corneal Anatomy and Homeostasis

The cornea is composed of 6 carefully organized layers that provide adequate structure and refractive power to the eye. From outermost to innermost, the cornea is formed by the epithelium, Bowman’s membrane, stroma, Descemet’s membrane, and the corneal endothelium. The corneal epithelium is a stratified (5–6 cell layers), non-keratinized squamous cell layer (~55–60 μm thick), serving as the external physical barrier of the cornea. Its basement membrane contains collagen types IV and VII, which help preserve surface integrity and promote continuous renewal. Bowman’s membrane is an avascular and acellular condensation of the superficial stroma (8–14 μm thick), composed of multiple collagen types (I, III, V, and VI), arranged in a dense network of fibers. It theoretically provides mechanical stability. The corneal stroma accounts for around 90% of the total corneal thickness (~550–640 μm thick). It is composed of highly ordered, parallel lamellae of collagen fibers (type I and V), containing sparse keratocytes (specialized fibroblasts) that maintain the matrix, and a ground substance rich in glycosaminoglycans (keratan sulfate) [12]. Their highly ordered structure, aided by proteoglycans and keratocytes, allows for transparency and tensile strength. More recently, the pre-Descemet’s layer (also known as Dua’s layer) was discovered. It is approximately 10–15 microns thick and contains compact collagen fibers, granting it increased tensile strength [13]. It consists of approximately 5–8 bands of type I and type IV collagen, and it is mostly acellular [13]. Descemet’s membrane, an elastic, acellular basement membrane (10–12 µm thick) composed of collagen types IV and VIII, provides a flexible yet resilient layer of the cornea [14]. Finally, the corneal endothelium is a single layer of hexagonal, squamous-to-cuboidal cells (5 µm thick) that maintains stromal deturgescence through Na^+^/K^+^ ATPase pumps. Its hexagonal arrangement allows for optimal adhesion, biomechanical support of the corneal tissue, and interaction with both the overlying stroma and the underlying Descemet’s membrane through specialized junctional complexes, including tight junctions and gap junctions. Human corneal endothelial cells are largely post-mitotic and lack significant regenerative capacity; therefore, cell loss, whether due to aging, trauma, or disease, results in compensatory enlargement and spreading of neighboring cells to maintain monolayer integrity and pump function [15].

The cornea is also host to a variety of specialized cells, which offer unique characteristics and contribute to corneal homeostasis and structure. Keratocytes are cells that synthesize and maintain ECM components, including collagen and proteoglycans [16]. When exposed to mechanical stress or injury, they can differentiate into fibroblasts and modify the ECM composition [17]. Specialized immune cells, including antigen-presenting cells (APCs) such as Langerhans cells and dendritic cells, reside in the peripheral zones of the cornea near the limbus and maintain active immune surveillance [18]. The upregulation of APCs has been shown during in vivo confocal microscopy examination in keratoconic corneas [19]. Other cells, including telocytes, help maintain stromal homeostasis and matrix integrity. Telocytes are interstitial cells characterized by an extremely small cell body and exceptionally long, thin cytoplasmic prolongations known as telopodes, which form an intricate network throughout the stroma. Through this network, telocytes establish close contacts with neighboring cells, including keratocytes, nerve fibers, and immune cells, facilitating intercellular communication, structural support, and paracrine signaling. Studies have shown that in KC, telocytes are diminished and display structural damage, suggesting that telocyte dysfunction may contribute to the disruption of stromal architecture characteristic of this condition. These findings point to telocytes as potential players in KC pathogenesis, highlighting their relevance as targets for future investigative and therapeutic approaches [20]. The cornea is also the most densely innervated tissue in the human body. Corneal nerves, originating primarily from the ophthalmic branch of the trigeminal nerve (cranial nerve V), enter the tissue at the limbus and traverse all corneal layers, forming a rich subbasal nerve plexus most prominently observed beneath the epithelium. These nerves are predominantly sensory, mediating pain, touch, and temperature, but also carry autonomic fibers that influence epithelial cell proliferation, keratocyte activity, and tear film secretion, a process collectively known as neurotrophic support. In particular, these nerves lose their myelin sheaths after about 1 mm of entry into the stroma [21]. Disruption of corneal innervation, whether due to surgery, infection, or systemic disease, can compromise epithelial integrity and healing, underscoring the critical role of neural input in corneal homeostasis [19,22]. Schwann cells (SCs) are important glial cells in the peripheral nervous system. In addition to providing structural support and myelination for axons, they play a vital role in peripheral nerve regeneration and tissue repair. Recent research has revealed a high concentration of both myelinating and non-myelinating SCs in the corneoscleral limbus. These limbal SCs are closely associated with limbal stem cells (LSCs) and are essential for regulating the homeostatic renewal and regeneration of the corneal epithelium. They often operate through paracrine signaling pathways to enhance LSC activity [23]. SCs also influence local protease and inflammatory activities in KC, thereby upregulating the local proteolytic environment [8].

The ECM of the cornea integrates both cellular and non-cellular components into a highly organized architecture that provides structural support and biomechanical strength, thereby conferring transparency, curvature, and refractive power to the tissue [1]. It is primarily composed of collagen fibers, proteoglycans, such as lumican, keratocan, decorin, mimecan, and glycoproteins, all of which contribute to the maintenance of stromal hydration and fibrillar organization [16,24]. These components are arranged into highly ordered lamellae, whose precise interfibrillar spacing and orthogonal orientation are essential for minimizing light scattering and ensuring optimal light transmission through the cornea.

ECM turnover is a tightly regulated process involving a balance between synthesis and degradation, mediated by interactions among matrix metalloproteinases (MMPs), tissue inhibitors of metalloproteinases (TIMPs), and lysyl oxidase (LOX). MMPs mediate collagen and proteoglycan turnover during wound healing and ECM renewal, driving controlled ECM degradation [25,26]. TIMPs counterbalance MMP activity, acting as regulatory checkpoints that ensure remodeling remains within physiological boundaries. LOX, in turn, participates in physiological corneal cross-linking (CXL) by catalyzing the conversion of lysyl residues on collagen into covalent CXL, thereby enhancing stromal stiffness, tensile strength, and resistance to deformation [27].

The cornea’s anatomy and ECM organization are sustained by a carefully maintained equilibrium among collagen synthesis, controlled proteolysis, and the coordinated interactions between keratocytes and immune mediators, a dynamic balance whose disruption underlies the pathogenesis of numerous corneal diseases.

## 3. Risk Factors and Main Pathophysiological Events Leading to Keratoconus

KC is a progressive, typically bilateral, although asymmetric corneal ectasia characterized by central or paracentral stroma thinning and protrusion into a cone shape. It is widely considered a multifactorial disease, likely caused by a combination of genetic predisposition and environmental factors, with oxidative stress and inflammation playing key roles in its pathogenesis and progression [28].

The clinical relevance of these risk factors has been recently validated at the highest level of expert agreement: a modified Delphi panel reaching unanimous consensus (100%) recognized eye rubbing and family history of KC as confirmed risk factors, with allergic eye disease (92%) and atopy (84%) also achieving consensus. Notably, dry eye disease (DED) did not reach consensus as a risk factor (53%), underscoring the ongoing debate regarding its independent contribution to KC pathogenesis [4,29].

The main pathophysiologic mechanisms underlying the development and progression of KC are numerous and varied. They can be categorized as (1) epithelial dysfunction and cytokine release; (2) increased degradative enzymes (proteolysis); (3) oxidative stress; (4) keratocyte apoptosis; (5) collagen disorganization and redistribution; (6) Bowman’s membrane breaks [30].

## 4. Biomechanical Alterations in KC

The biomechanical integrity of the cornea relies on a balanced interaction between its architecture, ECM, and viscoelastic properties. In KC, these biomechanical properties and their interactions become compromised. A structurally weak cornea is more susceptible to deformation, which can lead to progressive structural failure, ectasia, and the characteristic thinning and protrusion of the cornea [7,31].

The normal cornea exhibits viscoelastic behavior, allowing it to absorb and dissipate mechanical stress from external or internal sources, such as intraocular pressure, without permanently deforming [32]. This viscoelastic function combines elastic recoil and viscous damping. In KC, the viscoelastic property is disturbed. Alterations of the collagen network, reduced interfibrillar cohesion, and CXL lead to increased compliance and reduced resistance to deformation [33]. Recent studies have demonstrated that keratoconic corneas exhibit reduced stiffness, leading to lamellar slippage under stress [34]. The corneal stroma is also anisotropic, which allows the collagen lamellae to be preferentially oriented and, at the same time, confers direction-dependent mechanical strength. The loss of anisotropic orientation results in uneven deformation under stress and increases the risk of ectasia formation [35,36].

When a healthy cornea is subjected to mechanical stress, it exhibits a characteristic biphasic response: initial compliance, followed by nonlinear stiffening mediated by collagen fiber straightening and CXL [37,38]. In KC, this stiffening phase is attenuated, resulting in an altered stress–strain response characterized by greater compliance and a diminished elastic recovery [33,39]. Importantly, this biomechanical weakening is often focal rather than diffuse, consistent with the localized structural changes observed in early KC [40].

KC is therefore not merely a disease of corneal thinning and irregular topography, but a biomechanical disorder characterized by focal disruption of viscoelastic lamellar organization and localized loss of stromal stiffness. Under physiological stress, these focal zones of weakness cannot maintain normal load distribution, leading to progressive structural failure. This biomechanical framework not only underlies the pathophysiology of KC but also reinforces the importance of integrating biomechanical assessments alongside topographic indices for early detection and disease monitoring.

The clinical translation of these biomechanical concepts is increasingly supported by advanced in vivo technologies. High-speed dynamic Scheimpflug imaging (Corvis ST, Oculus Optikgërate GmbH, Wetzlar, Germany) and Brillouin microscopy have been explicitly recognized by the 2026 Global Consensus as validated tools for measuring corneal mechanical properties and resilience, with the potential to identify susceptibility to ectasia at preclinical stages [4]. Furthermore, the consensus identified the anterior radius of curvature as a more reproducible and repeatable metric than maximum keratometry (KMAX) for assessing disease progression, a finding directly relevant to understanding focal biomechanical failure in early KC. Indeed, 92% of panelists agreed that Kmax carries low repeatability, reinforcing the call for curvature-based parameters that better capture the heterogeneous and focal nature of biomechanical compromise in KC [4].

## 5. Oxidative Stress and Mitochondrial Dysfunction

**Figure 1:**
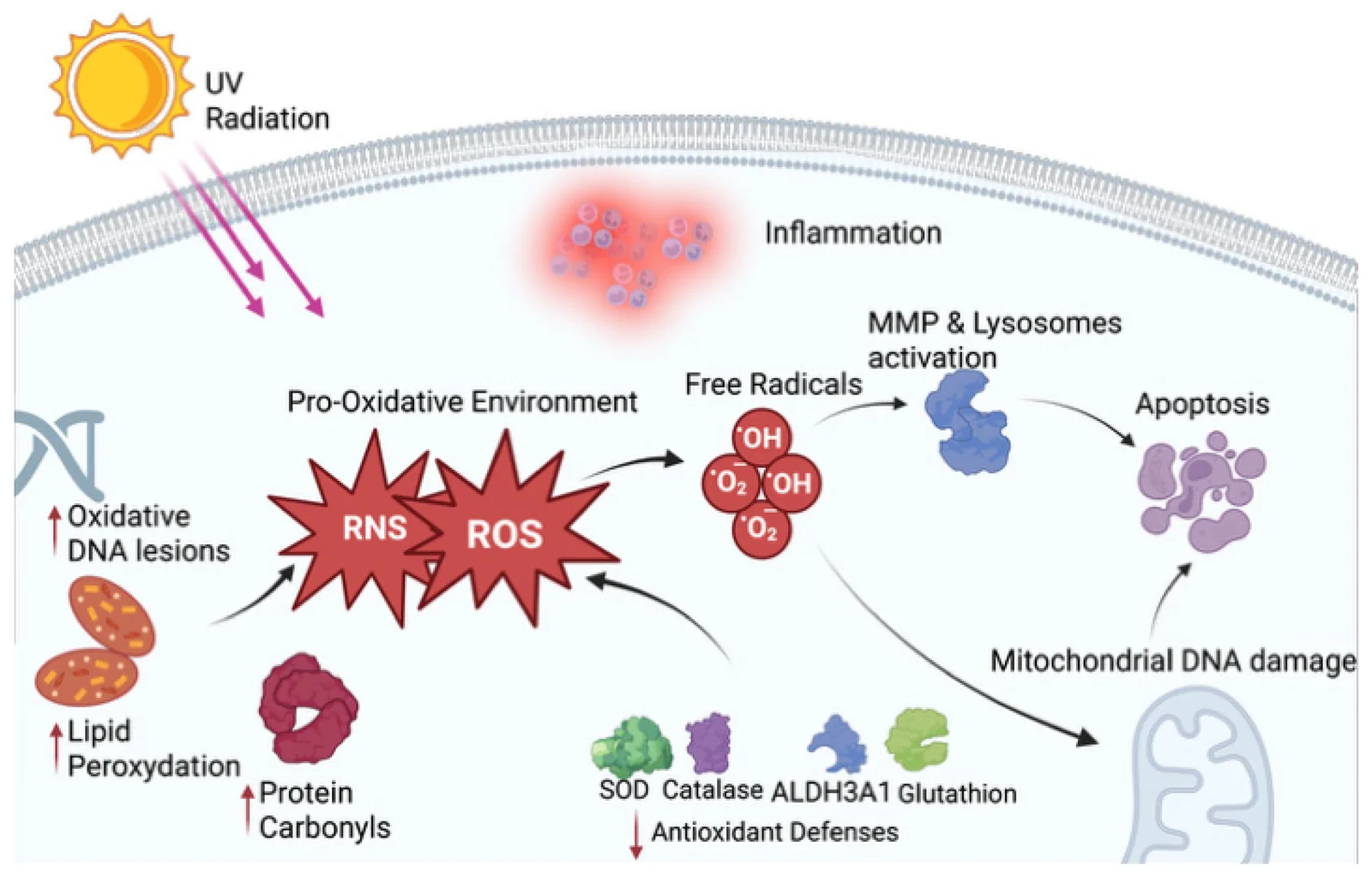
Oxidative stress pathway in KC. UV radiation, alongside increases in oxidative DNA lesions, lipid peroxidation, and protein carbonyls, and decreases in antioxidant defenses, generates increased ROS and RNS. This culminates in free radical formation, which directly damages mitochondrial DNA and upregulates MMPs and lysosomes, leading to keratocyte apoptosis.

Oxidative stress is among the most consistently implicated pathways in the pathogenesis of KC. The cornea is continuously exposed to environmental oxidants, including ultraviolet (UV) radiation, metabolically generated reactive oxygen species (ROS), and inflammatory mediators [41]. In healthy corneas, it maintains a balance with a robust antioxidant system. However, in KC, disturbances in this equilibrium create an accumulation of oxidative damage, mitochondrial dysfunction, and impaired ECM maintenance [41,42]. Figure 1 shows the oxidative stress pathway in KC.

Evidence supports that keratoconic corneas exhibit elevated levels of ROS and reactive nitrogen species (RNS) [43,44]. There is an increase in several byproducts, including lipid peroxidation, protein carbonyls, and oxidative DNA lesions, reflecting sustained oxidative injury [45]. Oxidative stress in KC is not confined to the cornea but has also been detected systemically, suggesting that the oxidative imbalance extends beyond local tissue damage and may reflect a broader systemic susceptibility in affected patients [46]. Concurrently, evidence indicates that keratoconic corneas exhibit depletion of major antioxidant defenses, including superoxide dismutase, catalase, aldehyde dehydrogenase (ALDH3A1), and glutathione, resulting in reduced expression and enzymatic activity [47,48]. This imbalance establishes a chronic pro-oxidative environment that weakens corneal structure and induces keratocyte apoptosis [49]. The resulting oxidative burden progressively compromises the cornea’s capacity to scavenge ROS and RNS, perpetuating cellular and stromal damage in a self-reinforcing cycle [50,51]. KC corneas also show signs of disturbed lipid peroxidation pathway byproducts. Accumulation of these oxidative stress byproducts can lead to apoptosis of corneal cells, especially keratocytes [52].

Mitochondria represent an additional significant source of ROS within the cornea [43,53]. In KC, mitochondrial deoxyribonucleic acid (DNA) undergoes substantial damage, including the accumulation of deletions and telomere shortening [54,55]. Elevated free radical levels can further exacerbate this damage, triggering mitochondrial stress, aberrant downstream signaling cascades, and ultimately cell death [43]. Keratocytes, as central regulators of corneal homeostasis, are particularly susceptible to mitochondrially mediated damage. This vulnerability is supported by in vitro evidence showing that stromal fibroblasts derived from KC corneas exhibit greater susceptibility to oxidative stress and a higher propensity for apoptosis than those from healthy tissue [56]. Furthermore, key proteins implicated in mitochondrial dysfunction, including GOT1 and TSTD1, which mediate oxidative stress-related cell death, have been identified in the context of KC [51].

An increase in ROS/RNS and subsequent overload can also have other consequences in the cornea, including an increased risk of KC. ROS/RNS can activate proteolytic pathways and increase the activity of MMPs and lysosomal proteases [42,55,57]. Likewise, in KC, TIMP downregulation due to increased ROS/RNS further contributes to ECM dysregulation [57,58]. This loss of keratocytes due to apoptosis and an increase in MMPs creates a negative ECM turnover. In addition, oxidative damage can impair CXL enzymes, specifically LOX. A dysfunction caused by increased oxidative imbalance further reduces collagen fiber cohesion and loosens stromal rigidity [59,60]. Oxidative stress in KC also disrupts normal autophagic pathways, as shown in KC epithelial cells, which exhibit altered marker expression under oxidative stress [56]. Yam et al. determined that the upregulated proteins in KC patients correlated with increased inflammatory activity and markers [51].

Prolactin-induced protein (PIP) is a glycoprotein expressed in several secretory tissues, including the lacrimal glands. PIP has been studied as a potential link between metabolic dysfunction and the pathophysiology of KC. PIP is significantly downregulated in tears, plasma, and saliva of patients with KC, supporting its role as a systemic and ocular biomarker of the disease [61]. Evidence suggests PIP may have a functional role in corneal metabolism, while KC fibroblasts demonstrate impaired ATP production from oxidative phosphorylation, and exogenous PIP treatment selectively restores glycolytic activity and mitochondrial respiratory capacity in these cells [62]. The findings suggest that PIP downregulation may contribute to altered bioenergetic and redox environments in KC keratocytes, impairing their ability to respond adequately to cellular stress and maintain stromal homeostasis.

The accumulated evidence indicates that free radicals and oxidative stress are core drivers of KC pathophysiology. ROS/RNS induce mitochondrial DNA damage, reduce antioxidant defenses, increase keratocyte apoptosis, accelerate ECM degradation, and overall undermine corneal structural integrity. These biochemical disruptions likely precede biomechanical failure and the clinical manifestations of KC.

## 6. Extracellular Matrix Remodeling

The structural integrity and mechanical resistance of the cornea rely upon a careful balance of matrix synthesis, CXL, and controlled degradation. In KC, this balance is disrupted. Increased matrix degradation, impaired CXL, and non-enzymatic modifications collectively compromise ECM stability.

As mentioned earlier, MMPs play a crucial role in ECM degradation and are tightly regulated by TIMPs. A reduction in TIMP-1 activity may increase gelatinase activity and contribute to apoptosis in the corneal stroma. It has been suggested that TIMP-1 inhibits the activity of MMP-2, which is the most significant protease found in the corneal stroma [63].

Previous studies have reported 1.9 times higher proteolytic activity and overexpression of several MMPs (MMP-1, -3, -7, and -13) in KC patients’ tears compared with controls [64]. Another study found a tendency toward overexpression of MMP-1, MMP-2, and MMP-9 in KC patients compared with healthy corneas [25,65]. Thus, lack of TIMP synthesis, coupled with increased MMP activity, creates a more degradative environment that, in turn, leads to progressive stromal thinning and weakening [66]. LOX and its isoforms catalyze the covalent formation of CXL between collagen and elastin fibers. In KC, studies have demonstrated reduced LOX expression and activity [67]. This reduction is observed in both the stroma and the epithelium of the cornea, suggesting a global deficiency in enzymatic CXL [68]. LOX-deficient KC corneas reduce the inter-fibrillar bonding and lower overall corneal stiffness, making it easier to deform under physiological or environmental stress [68,69].

The cornea is also subject to non-enzymatic CXL, many of which arise from glycation, oxidative adduct formation, or age-mediated molecular changes [69,70]. Glycation, the non-enzymatic process of binding sugar to proteins, leads to the accumulation of advanced glycation end products (AGEs) in KC, where they could contribute to disease formation [71]. This process is particularly relevant in patients with diabetes mellitus, who exhibit increased AGE deposition [69]. Nonetheless, AGEs may also exert a counterbalancing effect by increasing corneal stiffness, potentially offering partial protection against KC formation [69,72].

In KC, keratocytes exhibit exaggerated biological responses to stress stimuli that would normally induce only mild repair, leading to excessive apoptosis, abnormal cytokine release, and ECM remodeling [73,74]. Stromal keratocytes in KC patients have up to four times the number of interleukin (IL)-1 receptors than normal keratocytes, therefore heightening responses to injury and exhibiting increased susceptibility to cytokine signaling from IL-1 and others such as tumor necrosis factor alpha (TNF-α), and transforming growth factor beta (TGF-β) [75,76]. This subsequently induces keratocyte apoptosis from IL-1 pathways and signaling, further biomechanically weakening the corneal ECM [73,74]. This concept, regarded as cellular hypersensitivity, promotes mitochondrial dysfunction, reduces antioxidant defenses, and activates MMPs [73,76]. Corneal epithelium in KC also exhibits a heightened permeability to IL-1, which enhances the exposure of keratocytes to this IL [73]. Mixed responses to hypersensitive keratocytes in turn further impair ECM remodeling.

Several diverse factors related to corneal healing play key roles in KC development. Specifically, fibroblast growth factor 2 (FGF-2), platelet-derived growth factor (PDGF), and epidermal growth factor (EGF) are elevated in KC cases [77]. These factors are key modulators of KC development by influencing keratocyte proliferation, myofibroblast differentiation, and collagen production [78,79].

FGF-2 is involved in corneal wound healing. FGF-2 is released by epithelial and stromal cells following injury, causing keratocytes to differentiate into fibroblasts [18,80]. From the standpoint of ECM remodeling, FGF-2 can downregulate keratan sulfate proteoglycans (lumican and keratocan), which are crucial for maintaining corneal stromal transparency [81]. Furthermore, studies in FGF receptor 2 (FGFR2) knockout models have shown that loss of signaling leads to features mimicking KC, including localized central thinning, increased steepness, fibroblast activation, and higher rates of apoptosis [82].

PDGF is a potent mediator released by corneal epithelial cells and tears following injury [83]. Similar to FGF, PDGF stimulates the proliferation and migration of stromal keratocytes, promoting their differentiation into fibroblasts, a key component of the stromal response to injury [84]. Also, it has been shown that PDGF may be involved in the production of 62,000 MMP-2 species, which are abnormally produced by early-phase keratoconic corneal keratocytes [85]. EGF is also a major regulator of corneal epithelial wound healing and homeostasis [83]. In keratoconic corneas, EGF contributes to keratocyte activation, which can drive undesirable, disorganized remodeling that, in the context of impaired stroma, can lead to fibrosis [86].

In addition, overexpression of TGF-β, IL-1, vimentin, and tenascin-C has been observed in keratoconic corneas. TGF-β is a central regulatory cytokine in corneal homeostasis. Evidence in KC points to dysregulation of TGF-β, which strongly contributes to ECM balance, directly contributing to stromal thinning and disorganization, and altering keratocyte function [87,88]. TGF-β is the primary cytokine involved in collagen synthesis, keratocyte differentiation, and ECM architecture. In KC, the cornea can exhibit abnormal signaling and expression of TGF-β receptors, including TGFBR1 and TGFBR2, as well as dysregulated SMAD protein signaling [89]. This dysregulation leads to a defective wound-healing response and permits unchecked proteolysis and ECM remodeling [89,90]. Dysregulation of TGF-β can also induce keratocyte apoptosis or malfunction. In KC, it leads to loss of quiescent keratocytes, which, in turn, diminishes collagen production and impairs stromal maintenance [24,91].

Vimentin and tenascin-C play critical roles in corneal wound repair, particularly in the transition from normal healing to fibrosis [92,93]. Vimentin acts as a structural and signaling mediator for fibroblast activation and migration, while tenascin-C acts as a matricellular protein that modulates ECM to facilitate cell migration and wound closure; hence, the dysregulation of both these molecules often leads to corneal scarring [81]. Hence, deregulation of repair mechanisms in the cornea promotes a state of chronic damage and results in a weak reparative response to secondary injury (e.g., scratching, contact lens wear, oxidative and UV damage), suggesting that KC may be in a state of persistent injury, unable to heal due to a weakened repair response [90,94].

Furthermore, cytokines, including IL-1, TNF-α, TGF-β, IL-6, IL-8, and PDGF, regulate a cascade of proteases that affects the plasmin system, mainly tissue-type plasminogen activator (t-PA), urokinase-type plasminogen activator (u-PA), and plasminogen activator inhibitor. The plasminogen activator system is involved in corneal ECM remodeling, wound healing, and epithelial migration [95]. In KC, an imbalance in these proteolytic systems contributes to matrix degradation [96,97]. Finally, these same cytokines also activate prostaglandins (PGs) and MMPs, which ultimately alter the ECM of the keratoconic cornea [98,99,100].

In summary, ECM remodeling in KC involves a triad of pathological disruptions: elevated proteolytic activity driven by MMP overexpression and TIMP deficiency, deficient enzymatic CXL due to reduced LOX expression, and dysregulated non-enzymatic modifications, including glycation-derived AGE accumulation, whose net effect on corneal stiffness remains context-dependent. Also, keratocytes in KC have heightened sensitivity to IL-1, and healing factors in the cornea show important alterations in KC formation. Together, these mechanisms erode the structural and biomechanical integrity of the corneal ECM scaffold. It is also important to note that the degree of ECM remodeling may vary among individuals, likely reflecting an interplay of genetic susceptibility, local microenvironmental stressors such as eye rubbing, and behavioral, environmental, or age-related factors. Collectively, this active and multifactorial remodeling process is central to KC pathogenesis, representing a convergence of proteolytic, CXL, and cellular signaling dysregulation that progressively undermines corneal stability.

## 7. The Role of Inflammation in the Pathogenesis of KC

Historically, KC was defined as a degenerative non-inflammatory disease. The absence of clinically evident signs of inflammation reinforced this assertion. However, this conceptualization has been challenged by accumulating biomechanical and cellular evidence indicating chronic, low-grade inflammation and immune activation at the ocular surface in KC patients [98,101,102].

Multiple independent studies have reported an imbalance of pro-inflammatory cytokines in the tear film of KC patients. These include IL-6, IL-1β, TNF-α, and elevated levels of proteolytic enzymes such as MMPs [101,102,103]. Further tear proteomic and targeted assays expand on these findings, including elevations of multiple cytokines, such as those previously mentioned, and IL-17, IL-4, IL-5, IL-8, IL-10, IL-12, IL-13, IL-17, interferon (IFN)-γ, and chemokine C-C motif ligand 5 (CCL5) in KC [64,104,105]. These cytokines are usually elevated and then decrease after corneal CXL [105,106]. Levels of these enzymes or cytokines usually correlate with disease severity, with more advanced KC showing higher levels [107]. MMP-9 is usually elevated more severely in advanced KC, whereas IL-6 and TNF-α are elevated even in subclinical KC [66,101,102]. In diverse tissues, IL-1β and TNF-α have catabolic effects. In the cornea, they induce keratocyte apoptosis, inhibit collagen synthesis, and upregulate MMP transcription. These changes reduce stromal matrix and mechanical integrity [41].

Dysregulation of anti-inflammatory and immunomodulatory proteins has been identified in patients’ tears. For example, lactoferrin, immunoglobulin A (IgA), zinc-α2-glycoprotein (ZAG), and immunoglobulin κ-chain (IGKC) are decreased in patients with KC [90]. These molecules play an important role in modulating inflammation. Lactoferrin inhibits IL-1, IL-2, IL-6, and TNF-α, while IgA inhibits immune response through the IgA Fc receptor (FcαRI) [108].

Elevated levels of lysosomal cathepsin B and G have been found in keratocytes located beneath affected regions of Bowman’s membrane and in stromal regions with morphological changes in keratoconic corneas [109]. Elevated levels of cathepsin B and reduced levels of immunoglobulin-binding proteins, α-fibrinogen, cystatin SN, and cystatin S in the tears of patients with KC indicate an imbalance between proteases and their inhibitory molecules [110,111]. A study analyzing changes in the tear protein profile of KC patients found a significant reduction in cystatin, a natural inhibitor of cysteine proteinases, which are involved in the initial phases of intracellular protein degradation and can provoke tissue damage after being liberated into the ECM [111]. On the other hand, extracellular cystatins protect against the damaging effects of lysosomal proteinases, which are secreted under physiological conditions for tissue degradation and regeneration [112]. Therefore, the decreased levels of cystatins in KC tears reflect an increase in the degradation of total tear proteins found in KC tears.

Similarly, the decreased concentration of the anti-inflammatory tear proteins lipofilin-A and -C in KC patients, as reported by Versura et al. [113]. may lead to altered tear stability due to higher levels of free lipids in tears. When the lipid layer is abnormal, the tear film evaporates faster, leading to shorter tear break-up times with consequent epithelial cell damage. Similarly, decreased levels of phospholipase A2 in tear samples from KC patients could promote tear film instability by increasing the abundance of phospholipids in tears [113,114]. Contrary to these findings, serum albumin, a plasma-derived protein detectable in tears due to its leakage from conjunctival blood vessels, has been shown to increase by more than 3 times in keratoconic eyes compared to healthy controls, potentially representing a breach of the haemato-ocular barrier [111].

Another study investigated the relationship between the severity of KC, assessed using topographic indices, and tear inflammatory mediators that may contribute to KC inflammation and progression. The study measured the concentrations of IL-6, IL-13, IL-8, CCL-5, regulated upon activation, normal T cell expressed and presumably secreted (RANTES), MMP-9, MMP-13, TIMP-1, nerve growth factor (NGF), and EGF, using cytometric bead array technology [115]. The findings revealed significant positive associations between CCL5, MMP-13, and NGF with several topographic indices. Conversely, there were significant negative correlations between IL-6 and the keratoconus index (KCI). Additionally, age-dependent associations were noted between IL-13, IL-8, CCL5, and MMP-13 and the topographic data [115].

Paradoxically, altered levels of certain tear cytokines, alongside the downregulation of others, support part of the theory of parainflammation [116]. KC is not a characteristically inflammatory disease, as it lacks many changes characteristic of inflammatory disorders. The persistent imbalances of inflammatory cytokines and MMPs in the tear film of patients with KC suggest a constant chronic parainflammatory disease in which the tissue is continually affected [116]. KC can also develop in the context of chronic inflammatory conditions of the cornea. Patients who present with constant eye rubbing, either in severe atopic cases or ocular allergy disease. Just 4 h after constant eye rubbing, the number of neutrophils increases by over 2300%, while other cells, like degranulated mast cells, also increase in number [117]. This correlates with other studies in which the tear levels of MMP-13, IL-6, and TNF-α increase substantially after eye rubbing [118]. While KC usually develops in the context of parainflammation, it can coexist and develop in the context of inflammatory conditions of the cornea, especially in the presence of eye rubbing.

Finally, in vivo confocal microscopy (IVCM) further supports the presence of inflammation in corneas with KC. Studies have identified an elevated presence of corneal dendritic cells and Langerhans cells, and a lower stromal keratocyte density in patients with KC compared with healthy subjects [22,119].

Despite these findings, clinically, the keratoconic cornea appears uninflamed, lacking signs such as redness, edema, or pain [108,120]. Nonetheless, the mounting evidence supports low-level inflammatory changes in KC that alter homeostasis without evident inflammatory macroscopic changes [90].

## 8. Neurobiology of the Cornea in Keratoconus

The cornea’s innervation and sub-basal plexus offer more than tactile sensation to its structure.

It is also partially responsible for maintaining epithelial integrity, tear reflexes, and trophic support by releasing neuropeptides and neurotrophins [121]. In KC, IVCM has shown a reduced sub-basal nerve density, increased tortuosity, fragmentation, and abnormal morphology. These findings correlate with other imaging and diagnostic studies and spatially align with topographic and tomographic indices of ectasia [22,122,123]. Impaired corneal nerves lead to dysfunction in reflex tearing and blink efficiency, resulting in tear film instability and promoting epithelial microtrauma and chronic low-grade inflammation [124]. This environment promotes chronic microinjury, which is a cornerstone of KC pathophysiology.

Corneal nerves secrete important neurotrophic factors, including NGF, brain-derived neurotrophic factor (BDNF), and glial cell line-derived neurotrophic factor (GDNF). These factors are essential for supporting keratocyte survival and for maintaining epithelial homeostasis [125]. In KC, a reduction in these neurotrophic factors has been observed, which can lead to decreased stromal regenerative capacity and further destabilize the ECM [78]. NGF has also been associated with keratocyte loss and apoptosis in KC. When neurotrophic factors are imbalanced, keratocytes may become downregulated. As their density declines, collagen and proteoglycan synthesis is impaired, creating an environment that promotes ECM degradation [41,56]. This scenario links nerve dysfunction to a progressive reduction in the mechanical competence of the stroma.

Neuropeptides, amino acid chains that function as neurotransmitters, neuromodulators, or hormones, such as substance P, calcitonin gene-related peptide, and neuropeptide Y, modulate corneal epithelial migration, barrier function, and wound healing [126]. Substance P is involved in the regulation of collagen synthesis in corneal fibroblasts by promoting TGF-β signaling [127]. Dysregulation of neuropeptides can induce a pro-inflammatory state characterized by elevated cytokine levels, increased MMP activity, and a shift in the cornea toward a proteolytic environment [128,129]. This activity can contribute to KC development by degrading the ECM and reducing corneal CXL resistance. Dendritic cells in the cornea can also be activated by neurogenic inflammation, especially via nociceptive channels, such as transient receptor potential vanilloid-1 (TRPV1). These channels respond to inflammation and amplify immune responses, and can increase MMP-9 and other ECM-degrading enzymes [130,131].

Whether alterations in corneal neurobiology are a cause or consequence of KC remains to be determined. Nonetheless, the convergence of structural nerve plexus disruption, impaired neurotrophic support, and dysregulated neuropeptide signaling collectively amplifies ECM degradation, sustains a chronic sub-inflammatory state, and impairs keratocyte function, establishing corneal neurobiology as an active contributor to KC pathogenesis.

## 9. Genetic and Epigenetic Architecture

KC has long been recognized as a polygenic disease with a high genetic predisposition involving multiple genes and loci [132]. Familial aggregation studies support a medium-to-high heritability, with the prevalence of KC among first-degree relatives estimated at approximately 3.3%, between 15 and 67 times higher than the general population prevalence, depending on the population studied [133,134]. Multiple genes have been identified as candidate or genome-wide association study (GWAS) loci, including visual system homeobox 1 (VSX1), Zinc Finger E-Box Binding Homeobox 1 (ZEB1), Superoxide Dismutase 1 (SOD1), Zinc Finger Protein 469 (ZNF469), Dedicator Of Cytokinesis 9 (DOCK9), and Sodium bicarbonate transporter-like protein 11 (SLC4A11) [132].

Several genes are central to KC pathophysiological pathways, directly affecting the signaling and enzymatic mechanisms that underlie corneal homeostasis. Superoxide dismutase 1 (SOD1), for example, encodes the enzyme responsible for dismutating superoxide radicals; mutations in SOD1 lead to accumulation of superoxide radicals and peroxynitrite in the stroma [135]. Variants in the LOX gene have also been implicated in KC, with multiple associated loci identified across studies [136]. Additionally, several genes encode TIMPs, and dysfunction resulting from mutations in TIMP3, a genetic marker for KC-associated variants, leads to enhanced ECM degradation due to inadequate MMP regulation [137].

MicroRNAs (miRNAs) are epigenetic regulators implicated in ECM organization, apoptosis, oxidative stress, and wound healing, and have been shown to exhibit altered expression profiles in KC patients [138,139]. Potential downstream and altered miRNA profiles in KC, particularly those involved in ECM organization, oxidative stress, and wound healing [140]. Analysis of both epithelium and stroma of KC corneas demonstrated miRNA dysregulation in pathways involving keratocytes and ECM homeostasis [141]. By analyzing RNA profiles of KC, insights have been gained into miRNA alterations of signal transduction, antigen processing, and cellular homeostasis as possible biomarkers for KC [138,139]. DNA methylation is another epigenetic process in which methyl groups are added to DNA sequences to activate or deactivate them. Several gene promoters in KC undergo DNA methylation. A recent study by Kabza and collaborators identified 112 DNA-methylated regions, most of which overlapped with sensitive KC sites [142,143]. Further studies have conflicting evidence on DNA methylation. Another study by Nowak demonstrated that, although mitochondrial genes can be dysregulated, no differences in DNA methylation sequencing were identified between patients with and without KC. This finding suggests that DNA methylation could play an unclear role in KC pathophysiology [144].

In summary, the genetic landscape is polygenic, with multiple affected variants contributing to disease pathogenesis. Common risk alleles, familial variants, and epigenetic modulations, when combined with other risk factors, can trigger KC development. Future research must include multiethnic GWAS, whole-genome sequencing across multiple ethnicities, and multimodal imaging of endophenotypes to accurately identify KC-associated genetic variants [132].

## 10. Environmental and Behavioral Modifiers of Keratoconus

**Figure 2:**
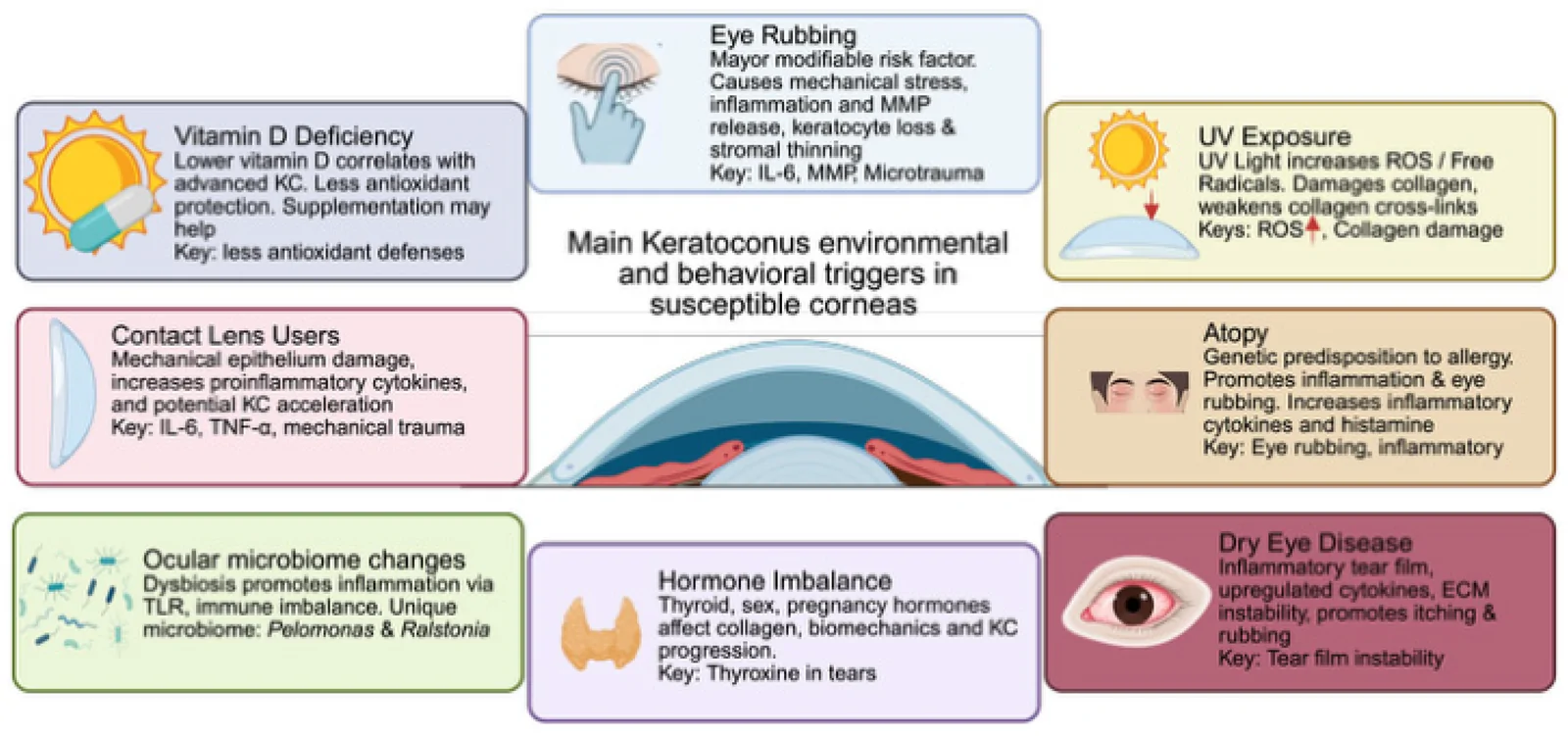
Main environmental and behavioral causes for KC formation. The major KC risk and causality factors. KC development arises from multiple pathways.

Multiple environmental and behavioral factors have been linked to the development of KC. While none of these factors alone is sufficient to cause the disease, they can act as mechanical or biochemical triggers in biomechanically or genetically susceptible corneas, precipitating or accelerating the pathological cascade underlying KC [145]. Figure 2 reviews the main triggers for KC development.

### 10.1. Eye Rubbing

Eye rubbing is the most implicated modifiable habit, considered a major risk factor for KC [124,146,147]. Eye rubbing and atopy can also be directly influenced by environmental factors, which can exacerbate these symptoms [148]. In study surveys, eye rubbing was present in a vast majority of patients: Assiri reported 44.8% [149], Rabinowitz reported 83% [150], and Leoni-Mesplie reported up to 91.8% in children with atopy [151]. Eye rubbing affects the cornea in multiple ways. As a viscoelastic tissue, the cornea is inherently susceptible to deformation under repeated mechanical stress [124,152]. The microtrauma caused by eye rubbing carries significant cellular consequences. Repeated mechanical injury to the epithelium and stroma promotes the release of pro-inflammatory cytokines and MMPs, depletes stromal keratocytes, and accelerates stromal thinning [153]. Mechanical deformation further disrupts keratocyte function and increases oxidative stress, perpetuating a self-reinforcing cycle closely associated with KC formation and progression [154,155]. Given this evidence, patient counseling on eye rubbing cessation represents a modifiable and clinically relevant intervention in KC management.

### 10.2. Ultraviolet (UV) Exposure

UV exposure is a source of ROS and can contribute to oxidative damage and the accumulation of free radicals in the cornea [145]. As previously mentioned, KC corneas exhibit dysfunction and reduced levels of enzymes that remove ROS/RNS. This correlates directly with higher KC prevalence across diverse countries. For example, countries in the Middle East, which geographically receive more sunlight than those in northern Europe, usually have a higher prevalence of KC [145]. Likewise, in vitro studies demonstrated that high UV exposure degrades corneal stromal collagen, induces focal thinning, and increases keratocyte loss [156]. However, UV may also act as a protective factor in some cases, as it induces corneal CXL, which, in turn, strengthens the corneal stroma against deformation [157]. Also, much of the hypothesis of UV exposure and free radicals rests on the higher prevalence observed in several sun-rich countries. Much of the discrepancy stems from these studies, which differ in how KC was diagnosed and the populations studied.

### 10.3. Atopy

Atopy refers to a genetic tendency to develop allergic diseases such as asthma, eczema, hay fever, and keratoconjunctivitis [9,145]. It is a significant risk factor for KC, often contributing through the release of inflammatory mediators due to chronic and vigorous eye rubbing [158]. Research indicates that over 50% of patients with KC may have allergies. Studies have established a strong link between atopic disorders and the progression of this condition [159]. Hypersensitivity reactions mediated by Immunoglobulin E (IgE) (type I) and cell-mediated delayed (type IV) are mainly involved in acute and chronic allergic disorders, respectively. Allergic conjunctivitis occurs when specific allergens, to which an individual has been sensitized, are exposed to the conjunctival-associated lymphoid tissue (CALT). These allergens bind to allergen-specific IgE on the surface of mast cells or basophils, provoking the release of inflammatory mediators [160]. These mediators include histamine, proteases, TNF-α, IFN-γ, platelet-activating factor (PAF), and diverse interleukins (i.e., IL-2, IL-4, IL-5, IL-13, IL-17), among others [160,161]. Additionally, an increase in proteases, protease activity, and inflammatory molecules in tears plays a significant role in the pathogenesis of KC [64]. As mentioned earlier, rubbing the eyes elevates the levels of tear MMP-13, IL-6, and TNF-α even in individuals without any underlying conditions [64,118]. It is hypothesized that this surge in protease activity may be further intensified by forceful eye rubbing commonly observed in patients with allergic conjunctivitis, consequently contributing to the development and progression of KC.

Mounting evidence supports an association between atopy and KC; however, isolating atopy as an independent risk factor remains methodologically challenging, as much of its contribution may be mediated through eye rubbing rather than through direct inflammatory mechanisms. Atopy likely functions as a facilitating condition that amplifies corneal vulnerability in genetically predisposed individuals, rather than as a direct cause of KC.

### 10.4. Dry Eye Disease

DED is one of the most frequent chief complaints in ophthalmology. Studies have shown that persistent DED alters proinflammatory cytokine levels in the tear film and ocular surface, thereby defining it as an inflammatory disease [162]. Upregulated cytokines in the tear film of patients with DED include IL-1β, IL-6, IL-8, IL-10, and TNF-α compared with healthy subjects [163,164]. As previously stated, mounting evidence considers KC an inflammatory condition. There is an association in KC patients with a higher incidence of DED risk factors, including Meibomian gland dysfunction (MGD) and blepharitis [165,166]. Patients with KC also have dysfunctional mucin and lipid layers of the tear film, further contributing to DED formation [165,166]. On examination, KC patients have lower tear break-up time (TBUT), non-invasive tear break-up time (NITBUT), tear meniscus height, and Schirmer–I test results, and higher ocular surface disease index (OSDI) scores when compared against controls [167,168]. Contact lens use also further increases dry eye symptoms, with reports referencing over 90% of patients with KC and contact lens use experiencing dry eye [169]. It has been suggested that elevated proinflammatory cytokines in DED might contribute to the development of KC, creating a proinflammatory local environment in which KC can further develop. The proinflammatory environment locally elevates MMP-9 levels, thereby creating ECM instability and accelerating degradation. DED also promotes itching, which can lead to eye rubbing by patients. Combined, DED promotes a proinflammatory state of the ocular surface, which can lead to ECM weakening or aggravating other triggers for KC.

### 10.5. Contact Lens Wear

Contact lenses have long been utilized for visual correction in patients with KC due to their irregular astigmatism, high refractive errors, and aberrations. A retrospective series from Macsai identified patients without KC who were fitted for contact lenses and were diagnosed with KC a mean of 12.2 years later [170]. Compared with non-contact use-related KC patients, they were older at diagnosis, had centered cones, and had flatter corneal curvatures [170]. Patients with prolonged contact lens use are prone to epithelial damage by mechanical injury, which releases apoptotic cytokines that play central roles in morphologic changes seen in patients with KC [171,172]. Contact lens use, especially rigid gas-permeable lenses, increases levels of proinflammatory cytokines (IL-6, TNF-α) and adhesion molecules such as ICAM-1 and VCAM-1 [172]. While contact lens use is key to the visual management of KC, it may also promote progression, and several cases have been associated with KC.

### 10.6. Changes in the Ocular Microbiome

Over the past decade, the study of the ocular surface’s microbiome has gained wide attention. The ocular surface microbiota comprises microorganisms that do not cause infection or inflammation and contribute to local homeostasis and immunological tolerance of ocular structures [173]. The ocular surface hosts a wide variety of microorganisms consisting of Gram-positive and Gram-negative bacteria, composed primarily of *Staphyloccocus*, *Corynebacterium*, *Streptococcus*, *Propionibacterium*, *Micrococcus*, *Haemophilus*, *Pseudomonas*, and *Neisseria* genera [174]. Metagenomic examinations have identified key core phyla and genera that are present in the conjunctiva of humans, including primarily Proteobacteria, Actinobacteria, and Firmicutes [175].

Recent studies have demonstrated changes in the ocular microbiome when compared to healthy controls in patients with KC. A study by Rocha-de-Lossada et al. identified key changes in microbiota, including the identification of two unique genera in KC: *Pelomonas* and *Ralstonia* [173]. Other studies have identified 8 more unique genera in KC while investigating reductions in key vital microbiota of the ocular surface of healthy patients, including *Sphingomonas* and *Atopobium* [176]. These changes in the ocular surface give way to dysbiosis, or microbial imbalance, of the ocular surface: commensal microorganisms are reduced or replaced by pathogenic ones. Dysbiosis, in turn, can lead to activation of Toll-like receptors, which can increase inflammatory responses of the ocular surface [59,174]. Kaur et al. found no significant differences in the ocular microbiome of the corneal epithelium between KC and healthy controls, which can in turn challenge the existing theories of dysbiosis of the ocular surface [59,177]. While the majority of studies point to ocular dysbiosis as a probable association for KC development, it cannot remain the sole factor. Alterations in the ocular surface microbiome may remain a key factor associated with KC development.

### 10.7. Hormone Imbalance

**Figure 3:**
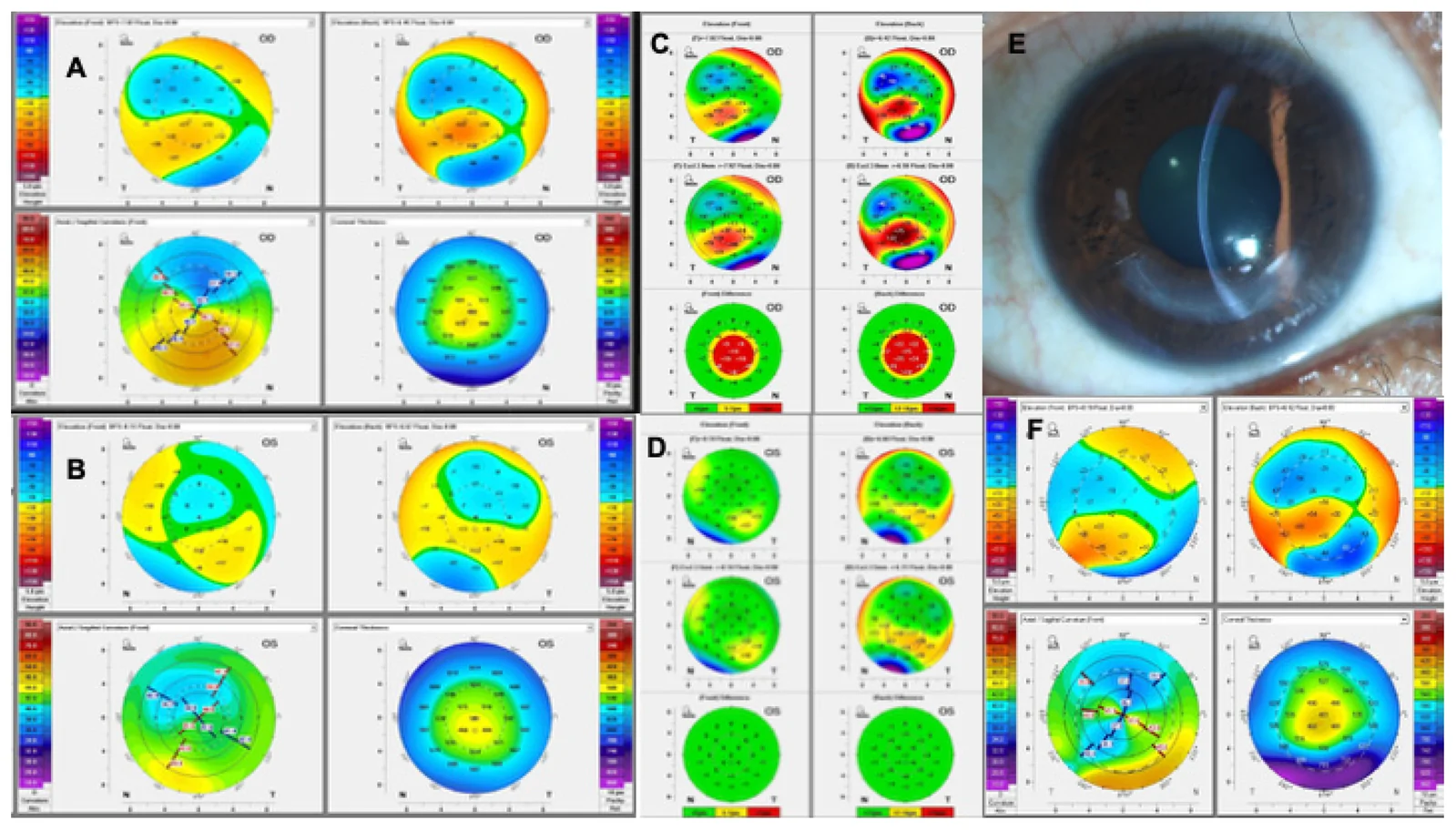
Case of KC development following pregnancy. A 23-year-old patient with a history of previous microkeratome laser in situ keratomileusis surgery who was pregnant 1 year ago arrived at the outpatient clinic complaining of blurry vision with spectacle glasses and changing vision. Pentacam examination revealed inferior corneal steepening and increased elevations in the right eye (**A**), which was more marked than the left eye (**B**). Berlin Ambrosio Enhanced Ectasia Display (**B**) demonstrated significant anterior and posterior differences in the right eye (**C**), while the left eye (**D**) maintained adequate values. The patient was counseled about potential treatment options. Corneal cross-linking was performed in both eyes, and corneal allogeneic intrastromal ring segments (CAIRS) were performed in the right eye with the implantation of a single inferior ring (**E**). The right eye showed improvement after surgery in inferior corneal steepening and regularity, with maximum keratometry decreasing from 48 to 43 following surgery (**F**).

Thyroid hormone imbalance has been increasingly associated with the development of KC. Thyroxine levels in the tear film are 3–4 times higher in KC patients with thyroid gland diseases than in normal controls. Thyroxine affects collagen production in the corneal stroma and supports KC pathophysiology [178]. KC development has also been associated with patients with secondary hyperthyroidism following radioactive iodine therapy and oral levothyroxine treatment, likely mediated by the abundance of thyroxine receptors in the cornea [179]. Changes in sex hormones have been theorized to influence KC development earlier and more aggressively in men, although these findings are challenging to reconcile across studies [180,181]. Hormonal changes in pregnancy can affect corneal biomechanical properties and thickness. KC has been shown to progress significantly during pregnancy, with hormonal changes potentially accelerating ectatic progression, particularly in newly diagnosed cases [9,182]. Figure 3 illustrates a case of KC development after pregnancy.

### 10.8. Vitamin D

Vitamin D has been associated with KC and has been shown to be decreased in patients with KC. Vitamin D acts as an antioxidant, helping protect tissues from inflammation. Patients with KC have lower serum 25-hydroxyvitamin D levels than control groups [183]. These levels also correlated with disease severity, with more advanced KC stages showing further decreased vitamin D levels. Furthermore, vitamin D supplementation has been suggested to aid with KC progression treatment [184].

## 11. Integrated Systems Model of Pathogenesis

**Figure 4:**
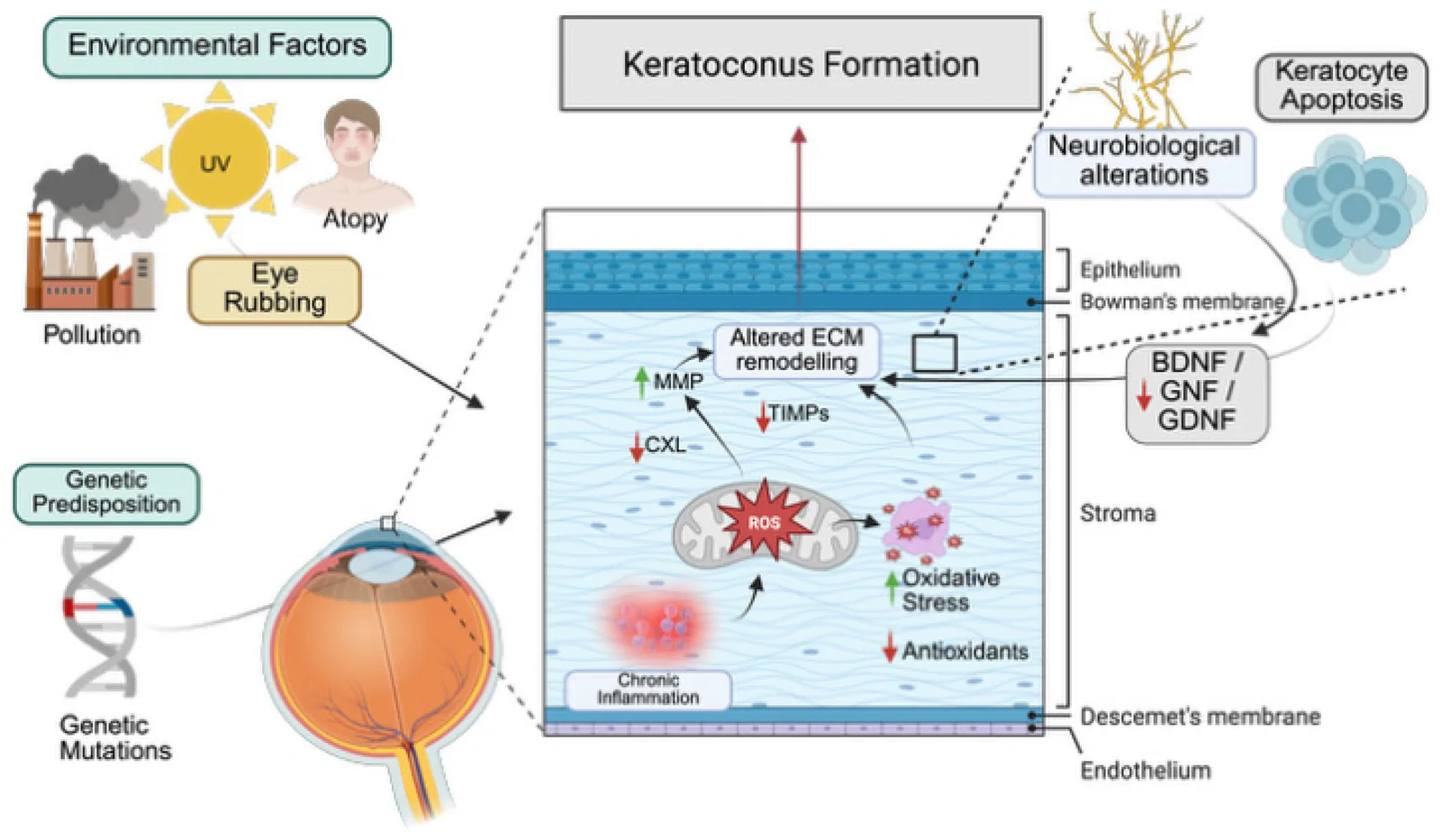
Major physiological pathways of KC development. Environmental factors, genetic predisposition, altered ECM remodeling, and cellular interactions, like neurobiological alterations and changes in keratocytes, converge in the formation of KC.

The pathophysiology of KC is understood not as a single pathway, but as an interaction of multiple systems. Figure 4 describes the major physiological pathways of KC. Biomechanical failure, impaired ECM remodeling, imbalance in proteolytic enzymes, free radical accumulation, aberrant immune signaling, genetic predisposition, and environmental factors all interact with each other to directly develop KC. At its core, KC requires stromal weakening, which allows for deformation and cone development. This is the key endpoint of all pathways and the main pathophysiological manifestation of the disease. Proteases and the enzymatic breakdown of the ECM, along with deficient CXL, can be the main drivers in the pathophysiology of KC. Genetic susceptibility, such as altered genomic profiles, is a key factor in the development of KC. Other pathways can alter the ECM; for example, accumulation of free radicals or neuroimmune aberrant signaling can cause effects at the ECM level. Other risk and causality factors, such as contact lens use, DED, and hormonal factors, play a role in KC formation, but it remains to be proven that there is a strong relationship. KC arises from a combination of interacting risk factors and pathogenic systems. Several hypotheses, including the “2-hit hypothesis” and the cascade hypothesis, converge on the view that multiple causal pathways can trigger KC formation. Table 1 summarizes the major changes in KC pathophysiology.

## 12. Future Directives and Conclusions

The pathophysiology of KC continues to represent a rich and evolving area of investigation. KC is a multifactorial disease shaped by the interplay of genetic predisposition, endogenous molecular changes, environmental exposures, and behavioral factors; a comprehensive understanding of these mechanisms is essential for advancing risk stratification, biomarker discovery, and therapeutic development. KC can develop through multiple pathways and risk factors that vary among patients, making it a complex process that can occur in different settings and contexts.

Future research should prioritize integrating biomechanics, cellular biology, neuroimmune signaling, and genomics in multiethnic cohorts, a critical need to validate existing findings and capture the full spectrum of genetic variability across populations. Emerging diagnostic modalities, including Brillouin microscopy, optical coherence elastography, RNA sequencing, spatial transcriptomics, and dynamic Scheimpflug imaging, promise to clarify the biomechanical origins of KC and enable earlier, more precise detection [186,187].

Novel therapeutics informed by the pathophysiological basis of KC may include neurotrophic interventions, ECM stabilizing agents, antioxidants, cell-based therapies, and gene therapy. However, most of these future therapeutic approaches remain in preclinical stages with limited evidence in human studies. Their clinical application remains uncertain, and further studies will be required before these can be incorporated into routine KC management.

## Figures and Tables

**Table 1:** Summary of molecular and receptor changes in KC.

Molecule/Receptor	Changes in KC	Role	Reference
Proteases & Inhibitors			
MMP	Increased	Overexpression in KC, enhances ECM degradation	[25,65,66]
TIMP	Decreased	Impaired inhibition of MMP-2, loss of ECM remodeling control	[63]
Cathepsin	Increased	Promotes stromal degradation inferior to Bowman’s membrane	[109]
Cystatin	Decreased	Increases tear protein degradation	[111]
CXL Enzymes			
LOX	Decreased	Deficient enzymatic CXL, reduces corneal stiffness	[27,67,68]
Antioxidants			
Superoxide dismutase (SOD)	Decreased	Impaired ROS degradation and accumulation of radicals	[47,48]
Catalase	Decreased	Reduced H_2_O_2_ neutralization, oxidative damage	[48]
ALDH3A1	Decreased	Reduced aldehyde detoxification, increased oxidative accumulation	[47]
Glutathione	Decreased	Depletes antioxidant defenses	[47]
Pro-inflammatory Cytokines in tear film			
IL-1 β, IL-6, TNF- α	Increased	Keratocyte apoptosis, inhibits collagen synthesis, upregulates MMP	[66,102,172]
IL-4, IL-5, IL-8, IL-10, IL-12, IL-13, IL-17	Increased	Cytokine imbalance in tear film, proinflammatory state	[64,104,105,108]
IFN- γ	Increased	Positively associated with increased KC in topographic indexes	[115]
MMP-9	Increased	Inflammatory marker and ECM instability	[66,102]
Anti-inflammatory/Immunomodulatory proteins			
Lactoferrin	Decreased	Reduced inhibition of IL-1, IL-2, IL-6, and TNF-alpha	[64,90]
IgA	Decreased	Impaired immune modulation via FcaRI receptors	[108]
ZAG/IGKC	Decreased	Reduced anti-inflammatory regulation of the tear film	[90]
Lipofilin-A	Decreased	Altered lipid tear film layer	[113]
Phospholipase A2	Decreased	Increased free phospholipids, promotes tear film instability	[113]
Albumin	Increased	Increased 3× against controls	[111]
Prolactin-Induced Protein	Decreased	Markedly decreased	[61,62]
Growth Factors			
FGF-2	Increased	Promotes keratocyte differentiation into fibroblasts	[18,77]
PDGF	Increased	Stimulated migration of keratocytes, promotes MMP-2 production	[77,85]
EDF	Increased	Disorganized ECM remodeling and fibrosis	[77,86]
TFG β	Dysregulated	Abnormal signaling, impairs wound healing	[88,89]
NGF	Decreased	Reduced neurotrophic support, keratocyte loss and ECM degradation	[56,78]
BDNF, GDNF	Decreased	Impaired keratocyte survival and epithelial homeostasis	[78]
Receptors			
IL-1 Receptors	Increased	Up to 4x more in KC keratocytes, heightened sensitivity to IL-1 signaling	[75,76]
TGFBR1/TGFBR2	Dysregulated	Abnormal expression and signaling, disrupts pathways and ECM homeostasis	[89]
Toll-Like Receptors	Increased	Promotes pro-inflammatory state and MMP-9	[185]
Structural/ECM proteins			
Vimentin	Increased	Promotes fibroblast activation and migration, corneal scarring	[81,93]
Tenascin-C	Increased	Modulates ECM for cell migration	[81,92]
Lumican, Keratocan	Decreased	Loss of corneal stromal transparency	[81]
Collagen CXL	Decreased	Reduced inter-fibrillar bonding, lower corneal stiffness	[68,69]
Systemic Markers			
Vitamin D (25-OH)	Decreased	Reduced Antioxidant and anti-inflammatory functions	[4,183]
Zinc, Copper, Selenium	Decreased	Pro-inflammatory and pro-oxidative systemic imbalance	[4]
ROS/RNS	Increased	Driver of mitochondrial damage, keratocyte apoptosis and ECM degradation	[43,44]
Mitochondrial damage	Increased	Deletions and telomere shortening, impairs cellular energy and promotes apoptosis	[54,55]

## Data Availability

No new data were created or analyzed in this study. Data sharing is not applicable to this article.

## Abbreviations

AGEs	Advanced Glycation End Products
APCs	Antigen-Presenting Cells
AI	Artificial Intelligence
BDNF	Brain-Derived Neurotrophic Factor
CCL5	Chemokine C-C Motif Ligand 5
CALT	Conjunctival-Associated Lymphoid Tissue
CAIRS	Corneal Allogeneic Intrastromal Ring Segments
CXL	Corneal Cross-Linking
DOCK9	Dedicator of Cytokinesis 9
DED	Dry Eye Disease
EGF	Epidermal Growth Factor
ECM	Extracellular Matrix
FGF-2	Fibroblast Growth Factor 2
GDNF	Glial Cell Line-Derived Neurotrophic Factor
GWAS	Genome-Wide Association Studies
IgA	Immunoglobulin A
IgE	Immunoglobulin E
FcαRI	IgA Fc Receptor
IGKC	Immunoglobulin κ-Chain
IFN-γ	Interferon Gamma
IL	Interleukin
IVCM	In Vivo Confocal Microscopy
KC	Keratoconus
KCI	Keratoconus Index
Kmax	Maximum Keratometry
LOX	Lysyl Oxidase
MMP	Matrix Metalloproteinase
MGD	Meibomian Gland Dysfunction
miRNA	Micro RNA
NGF	Nerve Growth Factor
NITBUT	Non-Invasive Tear Break-Up Time
OSDI	Ocular Surface Disease Index
PAF	Platelet-Activating Factor
PDGF	Platelet-Derived Growth Factor
PGs	Prostaglandins
PIP	Prolactin-induced protein
RNS	Reactive Nitrogen Species
ROS	Reactive Oxygen Species
RANTES	Regulated upon Activation, Normal T Cell Expressed and Presumably Secreted
SCs	Schwann Cells
SLC4A11	Sodium Bicarbonate Transporter-Like Protein 11
SOD1	Superoxide Dismutase 1
TBUT	Tear Break-Up Time
TIMP	Tissue Inhibitors of Matrix Metalloproteinases
t-PA	Tissue-Type Plasminogen Activator
TGF-β	Transforming Growth Factor Beta
TRPV1	Transient Receptor Potential Vanilloid 1
TNF-α	Tumor Necrosis Factor Alpha
UV	Ultraviolet
u-PA	Urokinase-Type Plasminogen Activator
VSX1	Visual System Homeobox 1
ZEB1	Zinc Finger E-Box Binding Homeobox 1
ZNF469	Zinc Finger Protein 469
ZAG	Zinc-α2-Glycoprotein
